# BCL11B expression in hepatocellular carcinoma relates to chemosensitivity and clinical prognosis

**DOI:** 10.1002/cam4.6167

**Published:** 2023-06-09

**Authors:** Hiroyuki Abe, Kenya Kamimura, Shujiro Okuda, Yu Watanabe, Jun Inoue, Yutaka Aoyagi, Toshifumi Wakai, Ryo Kominami, Shuji Terai

**Affiliations:** ^1^ Division of Gastroenterology and Hepatology, Graduate School of Medical and Dental Sciences Niigata University Niigata Niigata Japan; ^2^ Department of General Medicine Niigata University School of Medicine Niigata Niigata Japan; ^3^ Division of Bioinformatics, Graduate School of Medical and Dental Sciences Niigata University Niigata Niigata Japan; ^4^ Department of Agricultural Chemistry, Faculty of Applied Biosciences Tokyo University of Agriculture Tokyo Japan; ^5^ Department of Gastroenterology and Hepatology Niigata Medical Center Niigata Niigata Japan; ^6^ Division of Digestive and General Surgery, Graduate School of Medical and Dental Sciences Niigata University Niigata Niigata Japan; ^7^ Department of Molecular Genetics, Graduate School of Medical and Dental Sciences Niigata University Niigata Niigata Japan

**Keywords:** BCL11B, drug sensitivity, GATA6, hepatocellular carcinoma, prognosis

## Abstract

**Introduction:**

B‐cell lymphoma/leukemia 11B (BCL11B) is a subunit of SWI/SNF chromatin remodeling complexes and functions in cell cycle regulation and apoptosis upon DNA replication stress and damages via transcription. Many malignancies were reported to exhibit changes in BCL11B gene expression; however, no study has focused on the relationship between BCL11B and hepatocellular carcinoma, which potentially exhibits DNA replication stress and damages upon its oncogenesis. Thus, in this study, we examined the molecular characterization of BCL11B expression in hepatocellular carcinoma.

**Methods and Results:**

The cumulative progression‐free survival and overall survival were significantly longer in the clinical cases of BCL11B‐negative hepatocellular carcinoma than BCL11B‐positve cases. Microarray and real‐time PCR analyses in hepatocellular carcinoma cell lines indicated a correlation between BCL11B and GATA6, a gene reported to be correlated with oncogenic activities and resistance to anthracycline, which is often used for hepatocellular carcinoma chemotherapy. Consequently, BCL11B‐overexpressing cell lines exhibited resistance to anthracycline in cell growth assays and the resistance has been evidenced by the increased expression of BCL‐xL in cell lines. The results were supported by the analyses of human HCC samples showing the correlation between BCL11B and GATA6 expressions.

**Discussions and Conclusion:**

Our results indicated that overexpression of BCL11B amplifies GATA6 expression in hepatocellular carcinoma in vitro and in vivo that leads to anti‐apoptotic signal activation, and induces resistance to chemotherapy, which influenced the postoperative prognosis.

## Graphical abstract

B‐cell lymphoma/leukemia 11B (BCL11B) expression in hepatocellular carcinoma relates to chemosensitivity and clinical prognosis.

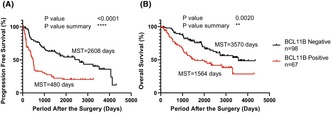

## 1 INTRODUCTION

The B‐cell lymphoma/leukemia 11B (BCL11B) gene encodes a lineage‐specific C2H2‐type zinc finger transcription factor protein. BCL11B expressed in various types of cells and contributes to the development of neuron, T cells, and others.^1^, ^2^, ^3^, ^4^, ^5^, ^6^, ^7^, ^8^, ^9^ Furthermore, BCL11B is known as an adipogenesis regulator^10^, ^11^ and a haploinsufficient tumor suppressor.^12^, ^13^, ^14^, ^15^ The BCL11B allele loss results in the occurrence of human T‐cell acute lymphoblastic leukemia and mouse thymic lymphoma.^1^, ^16^, ^17^ On the other hand, several studies have reported the effect BCL11B on cell proliferation and chemoresistance in cancer as an oncogene. For instance, BCL11B upregulation has been reported in Ewing sarcoma to maintain its oncogenic character^18^; the expression of BCL11B has been found to be correlated with the poorly differentiated tumor status of head and neck squamous cell carcinoma^19^; enriched expression of BCL11B in glioma cells promotes cell growth^20^; and BCL11B suppression using RNA interference technique inhibited leukemic T cells proliferation by apoptosis.^21^ This oncogenic mechanism might involve the apoptosis resistance accompanied by the delay of cell cycle by accumulating cells at G1 associated with the upregulation of p27, p57, and p18.^21^ The mechanism is also supported by the report that BCL11B contributes to the maintenance of genomic integrity at cell cycle as a result of the lack of the gene that causes the failure of checkpoint kinase 1 activity in thymocytes.^22^ These data support the fact that BCL11B‐overexpressing malignant cells may obtain resistance characteristics to chemotherapy and radiotherapy to induce apoptosis. As a fact, various cancer cells activate DNA repair pathways to gain chemo‐ and radio‐resistance characteristics.^23^, ^24^, ^25^ Based on this evidence, BCL11B could be a new therapeutic target in the refractory of malignancy to the conventional therapeutics.^26^, ^27^, ^28^

Hepatocellular carcinoma (HCC) is a malignancy related to DNA damage and mutagenesis caused by various hepatitis caused by virus, fatty infiltration, and alcoholic liver disease, which leads to carcinogenesis.^29^ Some studies have recently reported that DNA repair signaling pathway in HCC cells contributed to worse prognosis^30^, ^31^, ^32^, ^33^; however, no detailed mechanisms have been reported in this regard. Based on the role of BCL11B in biology, it is reasonable to hypothesize that BCL11B is involved in the clinical course of HCC as it is treated with various DNA‐damaging chemotherapy and radiotherapy agents. Regarding the relationship between BCL11B and HCC, only a few studies have reported BCL11B gene modification by mutation, deletion, amplification, truncation, gain, and copy number aberrations,^34^, ^35^ immune evasion mechanisms,^36^ and retention of cancer stem cell traits in HBV‐related HCC.^37^ Based on the aforementioned backgrounds, we have investigated the effect of BCL11B expression in HCC cells on resistance to various therapies and on HCC prognosis.

## 2 MATERIALS AND METHODS

### 2.1 Clinical course and BCL11B expression in vivo

With a written informed consent, tissue samples were collected from the HCC cases who were diagnosed with imaging studies including magnetic resonance imaging and computed tomography, and underwent surgical resection in Niigata University Hospital. The tissues were stained with hematoxylin and eosin or immunohistochemical stains: Rat anti‐Ctip2 (Bcl11b) antibody (ab18465; Abcam), Rabbit anti‐GATA6 antibody (ab175349; Abcam), Vectastain Elite ABC Rat immunoglobulin G kit (PK‐6104; Vector Laboratories), Vectastain Elite ABC Rabbit immunoglobulin G kit (PK‐6101; Vector Laboratories, Burlingame, CA), and 3,3′‐diaminobenzidine chromogen tablets (Muto Pure Chemicals). The expression of BCL11B and GATA6 was confirmed via RT‐PCR in recently resected 70 cases using the aforementioned procedure. The tumor tissues of these cases were immunohistochemically stained to determine the relationship between BCL11B and GATA6. Images were captured randomly, and analyzed quantitatively, using the ImageJ software (version 1.8.0_172; National Institutes of Health, Bethesda, MD).^38^

### 2.2 Microarray and bioinformatic analyses

Mock‐ and BCL11B‐transfected HLE cell lines were compared for the gene expression using SurePrint G3 Human Gene Expression (v2) Microarray Kit and GeneSpring GX version 14.5.1 (Agilent Technologies, Inc.). Among the 50,599 genes analyzed, 1052, which exhibited >2‐fold differences in expression, were assessed with related gene expressions using the Kyoto Encyclopedia of Genes and Genomes (KEGG) orthology database. The gene orthology terms were selected based on Fisher's exact test, followed by the Benjamini–Yekutieli correction method.

### 2.3 Cells

HLE and HepG2 cell lines were obtained from the Japanese Collection of Research Bioresources Cell Bank (National Institutes of Biomedical Innovation, Health and Nutrition, Ibaraki, Osaka, Japan). The cells were cultured in Minimum Essential Medium with fetal bovine serum (10%) and of penicillin and streptomycin (100 U/mL). The BCL11B complementary DNA was cloned in pCMV6‐Entry Tagged Cloning Vector (OriGene Technologies, Inc.). Either mock or BCL11B‐cloned vectors were transfected into the cells using FuGENE HD Transfection Reagent (Promega) and selected using G418 sulfate. Three independent clones were isolated from each of the two cell lines for the analyses.

### 2.4 Gene and protein expression in the cells

*BCL11B* and *GAPDH* genes expression was confirmed via reverse transcription‐polymerase chain reaction (RT‐PCR). RNA was prepared from the cells (RNA Easy Mini Kit) and the complementary DNA was synthesized from 1 to 5 μg of RNA (SuperScript II Reverse Transcriptase, Invitrogen). Complementary DNA products (1–2 aliquots) were used for PCR. The primers used are (F, forward; R, reverse):

1. BCL11B (F): CACTCATCCGTGATCACTTC
2. BCL11B (R): CGATGAGATTGCTCTGGAAC
3. GATA6 (F): GCGGGAGAGAGCACCAATC
4. GATA6 (R): GAGCCCATCTTGACCCGAAT
5. GAPDH (F): GGTCGGAGTCAACGGATTTGGTCG
6. GAPDH (R): CCTCCGACGCCTGCTTCACCAC

PCR protocol: 94°C for 10 min, followed by 30 cycles of (94°C for 30 s, 54°C for 30 s, and 72°C for 1 min) followed by 72°C for 7 min of extension.

The protein expression of BCL11B, BCL‐xL, BCL‐2, BAX, GATA6, and β‐actin was examined via western blotting. Cells were suspended in phosphate‐buffered saline and mixed with an equal volume of lysis buffer, tris–HCl (0.125 M, pH 6.8), 10% sucrose, 10% sodium dodecyl sulfate, 10% 2‐mercaptoethanol, and 0.004% bromophenol blue. The extract was subjected to gel (4%–15% Mini‐PROTEAN TGX Stain‐Free Protein Gels, Bio‐Rad Laboratories) and blotted onto Hybond membranes (GE Healthcare Life Sciences) and the bands were visualized (ECL plus Western Blotting Detection System, GE Healthcare Life Sciences).

Antibodies used were:

1. Rabbit anti‐Ctip2 (Bcl11b) antibody (ab28448; Abcam, Cambridge, UK)
2. Rabbit anti‐Bcl‐xL antibody (ab98143; Abcam)
3. Rabbit anti‐Bcl‐2 antibody (ab196495; Abcam)
4. Rabbit anti‐Bax antibody (ab104156; Abcam)
5. Rabbit anti‐GATA6 antibody (ab175349; Abcam)
6. Rabbit anti‐β‐actin antibody (ab8227; Abcam)
7. Anti‐rabbit immunoglobulin G horseradish peroxidase (NA934‐1ML; GE Healthcare Life Sciences, PA, USA)

Expression of BCL11B protein in the cells was confirmed immunohistochemically, using Rat anti‐Ctip2 (Bcl11b) antibody (ab18465; Abcam), Vectastain Elite ABC Rat immunoglobulin G kit (PK‐6104; Vector Laboratories), and 3,3′‐diaminobenzidine chromogen tablets (Muto Pure Chemicals).

### 2.5 Cell growth assay

The cells were plated in 96‐well tissue culture dishes at a concentration of 2 × 10^4^ cells per well in 100‐μL medium. Then, they were treated with CDDP or epirubicin using doses determined based on previously reported studies.^39^, ^40^ The cell growth was assessed using the Premix WST‐1 Cell Proliferation Assay System (Takara Inc.).

### 2.6 Statistical analyses

Data were analyzed by paired *t‐*test or one‐ or two‐way ANOVA followed by Bonferroni's multiple comparison test. The cumulative PFS and OS curves were generated by the Kaplan–Meier method, and the occurrence rates were compared using a log‐rank test. GraphPad Prism 9 software (version 9.3.1; GraphPad) was used for the analyses. *p* < 0.05 was considered statistically significant.

## 3 RESULTS

### 3.1 Effect of BCL11B expression in HCC tumor on prognosis

**FIGURE 1:**
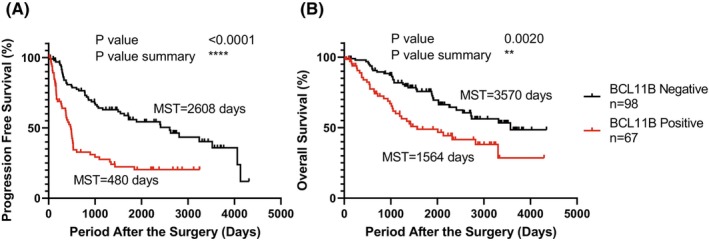
Effect of BCL11B expression on the prognosis of HCC. Kaplan–Meier estimates of the progression‐free survival (A) and overall survival (B) following the surgical treatment for HCC. MST, median survival time. **p < 0.01, ****p < 0.0001.

To examine the effect of BCL11B expression in HCC tumor cells on the prognosis of the reported cases, the PFS and OS were assessed in BCL11B‐positive (>5% positive in IHC analyses, *n* = 67) and BCL11B‐negative (*n* = 98) groups. The characteristics of the patients and their clinical information are summarized in Table 1. No significant differences were observed in age, gender, body mass index, etiology of the liver disease, histological classification of HCC, complication of liver cirrhosis, hepatic reserve function, tumor markers, nutrition, and number of postoperative treatments. The PFS (2608 vs. 480 days, *p* < 0.0001) and OS (3570 vs. 1564 days, *p* < 0.01) were significantly longer in the BCL11B‐negative tumor cell group (Figure 1). These results indicated that the BCL11B expression in HCC helps gain resistance to various postoperative therapeutic options and leads to poor prognosis.

**TABLE 1:** Patient characteristics.

BCL11B	−	+	MWW Test
	*n* = 98	*n* = 67	*p*‐value
Characteristics			
Age (years)			0.39
Median	65.5	69.0	
Range	35–82	34–82	
Gender			0.09
Female	23	24	
Male	75	43	
Body mass index			0.87
Median	22.7	23.1	
Range	16.9–31.6	14.9–34.2	
Etiology			0.11
HBV infection	29	12	
HCV infection	27	19	
Alcoholic	14	9	
Nonalcoholic steatohepatitis	23	25	
AIH, PBC	5	2	
Histology			0.93
Well‐differentiated tumor	29	21	
Moderately differentiated tumor	57	36	
Poorly differentiated tumor	12	10	
Cirrhosis			0.14
Yes/No	42/56	21/46	
Child–Pugh Score			0.80
5/6/7/8	74/19/3/2	51/10/5/1	
Stage			0.37
I/II/III/IV	23/34/24/17	16/15/22/14	
AFP (ng/mL)			0.19
Median	8.3	12.3	
Range	0.0–110,000	0.0–164,224	
DCP (mAU/mL)			0.21
Median	27.8	37.0	
Range	0.0–4340	0.0–6472	
TC (mg/dL)			0.20
Median	171.0	187.0	
Range	94.0–441.0	106.0–260.0	
TG (mg/dL)			0.53
Median	100.0	107.0	
Range	29.0–402.0	36.0–343.0	
HbA1c (%)			0.87
Median	5.5	5.5	
Range	4.1–9.7	5.0–8.1	
Postoperative Treatments (Number of Options)			0.06
0/1/2/3/4/5/6	17/3/9/7/2/1/1	6/6/16/4/10/0/0	

*Note*: The values are expressed as median and range. MWW test, Mann–Whitney‐Wilcoxon test.

Abbreviations: AFP, alpha fetoprotein; AIH, autoimmune hepatitis; DCP, des‐γ‐carboxy prothrombin; HBV, hepatitis B virus; HCV, hepatitis C virus; PBC, primary biliary cholangitis; TC, total cholesterol; TG, triglyceride.

### 3.2 Development of *BCL11B* ‐overexpressing HCC cells

**FIGURE 2:**
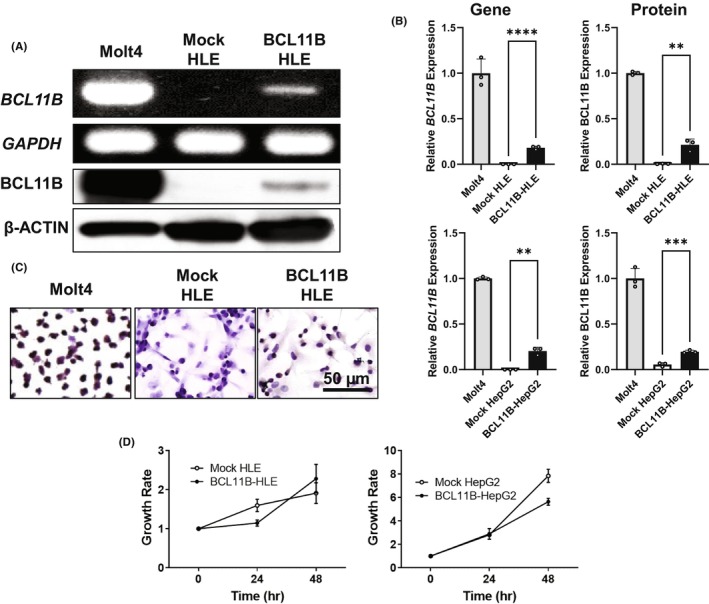
Development of BCL11B‐overexpressing hepatocellular carcinoma (HCC) cell lines. (A) Representative images of the reverse transcription‐polymerase chain reaction (RT‐PCR) of *BCL11B* and *glyceraldehyde 3‐phosphate dehydrogenase* (*GAPDH*) as well as Western blotting of BCL11B and β‐Actin in BCL11B‐overexpressing HLE. (B) Summary of relative expression ratios of the BCL11B gene and protein. The values are expressed as means ± standard deviations (*n* = 3), **p < 0.01, ***p < 0.001, ****p < 0.0001 on Student's *t*‐test. (C) Representative immunohistochemical staining of BCL11B‐overexpressing HLE. The T lymphoblast cell line of Molt4 was shown as a positive control, and the Mock‐transfected HLE was used as a negative control. (D) Growth of BCL11B‐overexpressing cell lines under a normal culture condition with 10% fetal bovine serum. Mock HLE and Mock HepG2 indicate Mock‐transfected HLE and HepG2 cell lines. BCL11B‐HLE and BCL11B‐HepG2 indicate BCL11B overexpression.

To examine the molecular function of BCL11B on the viability of HCC, we produced *BCL11B*‐overexpressing cell lines by transfecting plasmid DNA‐expressing human *BCL11B* into HCC cell lines, that are, HLE and HepG2. Figure 2A presents the *BCL11B* gene and BCL11B protein expression in the cells comparing with that of Molt4 cell line, which is known to express BCL11B. Quantitative analyses of multiple clones revealed a significantly higher expression of BCL11B in HLE and HepG2 cells by RT‐PCR, WB, and IHC (Figure 2B,C). Under normal cell culture condition with 10% fetal bovine serum, *BCL11B*‐overexpressing cells exhibited no significant difference in growth rate compared with the mock‐transfected cells (Figure 2D).

### 3.3 Effect of *BCL11B* expression on gene expression modification

**FIGURE 3:**
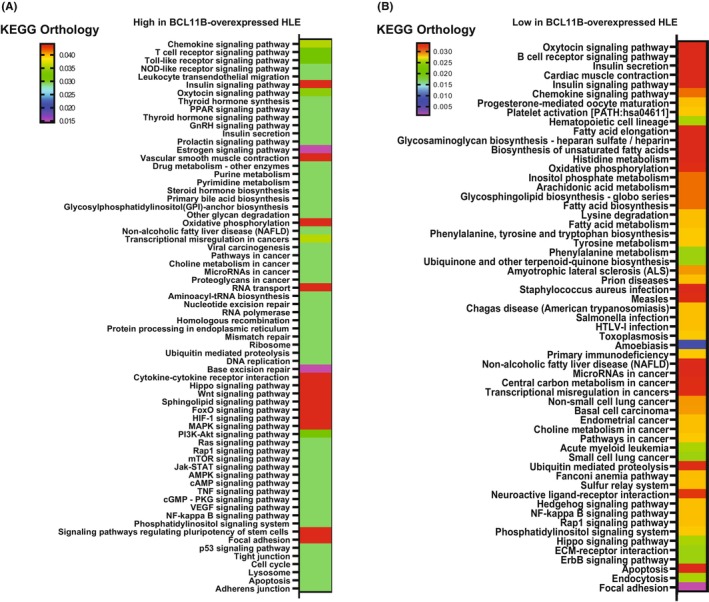
Microarray and bioinformatic analyses. KEGG orthology classification for the genes with more than 2‐fold differences in the expression. Higher (A) and lower (B) expressions in *BCL11B*‐overexpressing HLE than mock‐transfected HLE.

**FIGURE 4:**
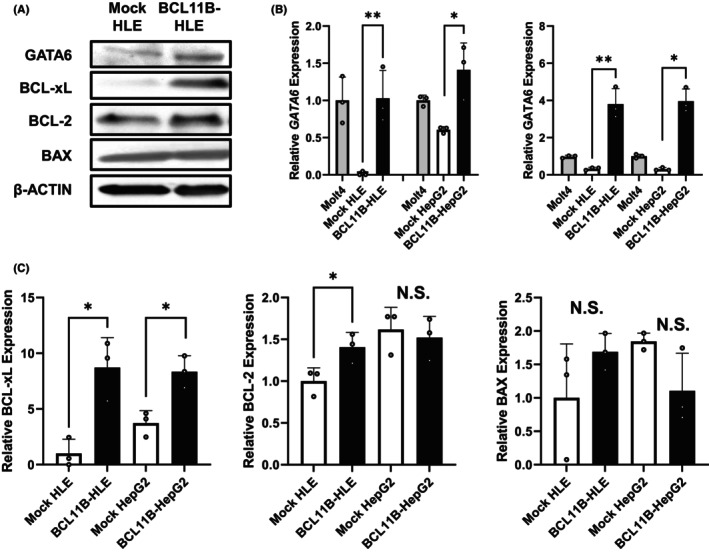
Effect of BCL11B expression on the changes in GATA6 and apoptosis‐related proteins in HCC cells. (A) Representative Western blotting images of GATA6, BCL‐xL, BCL‐2, BAX, and β‐Actin proteins in the cells. (B) Relative expression ratio of the GATA6 gene and protein expression. Molt4 cell was used as a positive control. (C) Relative expression ratios of the BCL‐xL, BCL‐2, and BAX proteins. The values are expressed as means ± standard deviations (*n* = 3), *p < 0.05, **p < 0.01, and N.S., not significant on Student's *t*‐test.

To determine the molecular mechanism of BCL11B, the gene expressions in mock‐transfected HLE and *BCL11B*‐overexpressing HLE were compared via DNA microarray analyses (Figure 3A,B). Analysis of KEGG orthology terms, after the hierarchical clustering of genes, revealed higher gene expression in terms of various oncogenic pathways, including the Jak–STAT, MAPK, Wnt, and Hippo signaling pathways, apoptosis, cell cycle, p53 signaling, and DNA replication in *BCL11B*‐overexpresing cells than in mock‐transfected cells. The genes upregulated and downregulated in *BCL11B*‐overexpressing cells are presented in Tables 2 and 3. Based on the analyses, in order to examine the mechanisms of BCL11B on prognosis of HCC cases, we have focused on the correlation of GATA binding protein 6 (GATA6), which showed 78905.9‐fold increase upon BCL11B‐overexpression, and apoptosis‐related gene expression. Moreover, GATA6 has been reported to be related to the differentiation and progression of the malignant disease, such as pancreatic cancer, colorectal carcinoma, and HCC.^41^, ^42^, ^43^, ^44^, ^45^, ^46^ The BCL11B‐overexpressing HLE and HepG2 cell lines demonstrated a significantly higher GATA6 expression than the mock‐transfected cells (Figure 4A,B). Furthermore, the BCL11B expression contributed to BCL‐xL increase in both cell lines and BCL‐2 increase in HLE but not to BAX expression. Altogether, these results indicated that BCL11B overexpression in HCC cells contributes to the induction of GATA6 expression and anti‐apoptotic phenotype (Figure 4C).

**TABLE 2:** Upregulated genes in *BCL11B*‐overexpressing HCC cell (>10‐fold differences).

No	Gene symbol	Gene name	Fold change BCL11B‐HLE versus Mock‐HLE
1	GATA6	GATA binding protein 6	78905.875
2	TMCO6	Transmembrane and coiled‐coil domains 6	306.944
3	CDHR2	Cadherin‐related family member 2	212.895
4	MAGEA9	Melanoma antigen family A, 9	41.670
5	LOC148696	Uncharacterized LOC148696	39.657
6	SEPW1	Selenoprotein W, 1	19.457
7	MTERF	Mitochondrial transcription termination factor	18.677
8	FAM75A2	Family with sequence similarity 75, member A2	17.510
9	FAM95B1	Family with sequence similarity 95, member B1	17.469
10	FGD6	FYVE, RhoGEF and PH domain containing 6	16.954
11	POLR2M	Polymerase (RNA) II (DNA directed) polypeptide M	16.948
12	ASMT	Acetylserotonin O‐methyltransferase	16.843
13	TIAM2	T‐cell lymphoma invasion and metastasis 2	16.720
14	SNORD115‐5	Small nucleolar RNA, C/D box 115–5	16.460
15	VWA5B1	von Willebrand factor A domain containing 5B1	15.905
16	MAP3K4	Mitogen‐activated protein kinase kinase kinase 4	15.575
17	DGKE	Diacylglycerol kinase, epsilon 64 kDa	14.888
18	ARV1	ARV1 homolog (S. cerevisiae)	13.988
19	PHLDB3	Pleckstrin homology‐like domain, family B, member 3	13.848
20	EIF2AK2	Eukaryotic translation initiation factor 2‐alpha kinase 2	13.694
21	TSPAN11	Tetraspanin 11	13.656
22	TRH	Thyrotropin‐releasing hormone	13.646
23	SERGEF	Secretion regulating guanine nucleotide exchange factor	13.439
24	PMP2	Peripheral myelin protein 2	13.399
25	PPP1R14D	Protein phosphatase 1, regulatory (inhibitor) subunit 14D	13.336
26	ADAMTS15	ADAM metallopeptidase with thrombospondin type 1 motif, 15	13.192
27	EFHC2	EF‐hand domain (C‐terminal) containing 2	12.900
28	TAF1C	TATA box binding protein (TBP)‐associated factor, RNA polymerase I, C, 110 kDa	12.747
29	SP3P	Sp3 transcription factor pseudogene	12.467
30	CENPN	Centromere protein N	12.437
31	UHMK1	U2AF homology motif (UHM) kinase 1	11.935
32	HSDL1	Hydroxysteroid dehydrogenase like 1	11.839
33	ENTPD3	Ectonucleoside triphosphate diphosphohydrolase 3	11.767
34	ADAM9	ADAM metallopeptidase domain 9	11.357
35	CDC7	Cell division cycle 7 homolog (S. cerevisiae)	11.190
36	PTPN4	Protein tyrosine phosphatase, non‐receptor type 4 (megakaryocyte)	11.044
37	KCNMA1	Potassium large conductance calcium‐activated channel, subfamily M, alpha member 1	10.926
38	RECQL5	RecQ protein‐like 5	10.898
39	RAB2B	RAB2B, member RAS oncogene family	10.835
40	MSL3	Male‐specific lethal 3 homolog (Drosophila)	10.752
41	SPAG6	Sperm associated antigen 6	10.731
42	TDRD1	Tudor domain containing 1	10.634
43	VSIG10L	V‐set and immunoglobulin domain containing 10 like	10.551
44	IAH1	Isoamyl acetate‐hydrolyzing esterase 1 homolog (S. cerevisiae)	10.509
45	UCK2	Uridine‐cytidine kinase 2	10.399
46	HKR1	HKR1, GLI‐Kruppel zinc finger family member	10.398
47	SOHLH2	Spermatogenesis and oogenesis specific basic helix–loop–helix 2	10.364
48	NFIA	Nuclear factor I/A	10.312

**TABLE 3:** Downregulated genes in *BCL11B*‐overexpressing HCC cell (>10‐fold differences).

No	Gene symbol	Gene name	Fold change BCL11B‐HLE versus Mock‐HLE
1	TCP11L2	T‐complex 11 (mouse)‐like 2	41.370
2	TRAPPC9	Trafficking protein particle complex 9	22.601
3	NLRC3	NLR family, CARD domain containing 3	22.437
4	ADAM5P	ADAM metallopeptidase domain 5, pseudogene	21.112
5	SPATA19	Spermatogenesis associated 19	19.573
6	PML	Promyelocytic leukemia	17.612
7	WDR31	WD repeat domain 31	16.985
8	ZNF319	Zinc finger protein 319	16.857
9	KLK14	Kallikrein‐related peptidase 14	15.325
10	HSBP1L1	Heat shock factor binding protein 1‐like 1	15.132
11	AREG	Amphiregulin	14.431
12	FTLP10	Ferritin, light polypeptide pseudogene 10	14.351
13	GOLGA6L6	Golgin A6 family‐like 6	14.257
14	CEACAM3	Carcinoembryonic antigen‐related cell adhesion molecule 3	13.712
15	HTR2C	5‐hydroxytryptamine (serotonin) receptor 2C	13.123
16	FAM207A	Family with sequence similarity 207, member A	12.942
17	ABLIM1	Actin binding LIM protein 1	12.457
18	DHRS4L2	Dehydrogenase/reductase (SDR family) member 4 like 2	12.452
19	MGC39372	Serpin peptidase inhibitor, clade B (ovalbumin), member 9 pseudogene	12.266
20	THNSL2	Threonine synthase‐like 2 (S. cerevisiae)	12.248
21	CPLX4	Complexin 4	11.574
22	MBD1	Methyl‐CpG binding domain protein 1	11.509
23	ALK	Anaplastic lymphoma receptor tyrosine kinase	11.051
24	FPR1	Formyl peptide receptor 1	10.717
25	RPUSD2	RNA pseudouridylate synthase domain containing 2	10.500
26	AHNAK	AHNAK nucleoprotein	10.249
27	ZNHIT1	Zinc finger, HIT‐type containing 1	10.037

### 3.4 Effect of *BCL11B* expression on cell growth under a cytotoxic condition

**FIGURE 5:**
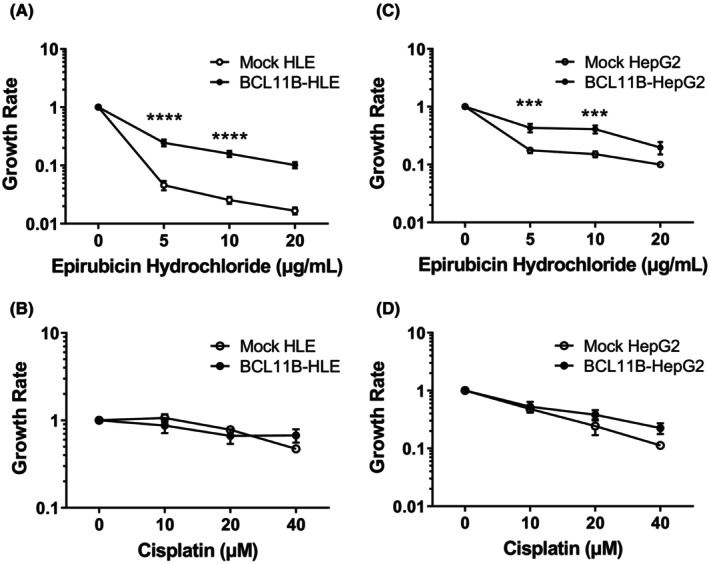
Effect of BCL11B expression on HCC cell growth. Cell growth rate of permanent clones of mock‐transfected and BCL11B‐overexpressing HCC cell lines determined via WST‐1 assay. HLE (A, B) and HepG2 (C, D) cells 24 h after culture in the medium with epirubicin hydrochloride (A, C) and cisplatin (B, D). The values are expressed as means ± standard deviations (*n* = 9, triplet with three clones), ***p < 0.001, ****p < 0.0001 on Student's *t*‐test.

To examine the effect of BCL11B on cell viability under a cytotoxic condition, cell growth assay was conducted using *BCL11B*‐overexpressing cells exposed to either epirubicin hydrochloride or cisplatin, which are often used in chemotherapy for HCC (Figure 5). While mock‐transfected HLE and HepG2 cell lines exhibited dose‐dependent cell growth inhibition when treated with epirubicin hydrochloride (Figure 5A,C) or cisplatin (Figure 5B,D), *BCL11B*‐overexpressing HLE and HepG2 showed a significantly milder inhibition with epirubicin hydrochloride (Figure 5A,C). These results indicated that BCL11B overexpression induced resistance to epirubicin hydrochloride in HCC cells.

### 3.5 Expression of BCL11B and GATA6 in HCC tissues

**FIGURE 6:**
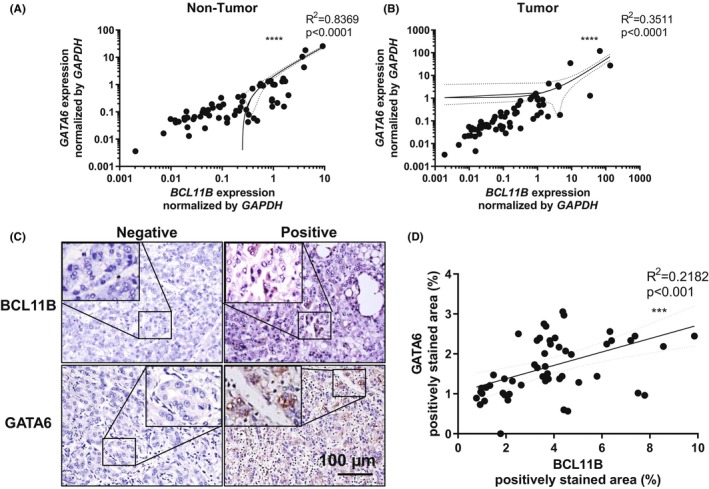
BCL11B and GATA6 expressions in HCC tissues. Relationship between the *BCL11B* and *GATA6* gene expressions in the tissues. (A) non‐tumor tissue surrounding HCC and (B) HCC tissue. (C) Representative images of the immunohistochemical staining of BCL11B and GATA6 in HCC tumors. (D) Relationship between the BCL11B‐ and GATA6‐stained areas (*n* = 70), **p < 0.01, ***p < 0.001, ****p < 0.0001, Pearson's correlation test. *r*, correlation coefficient. Continuous lines are the best hit lines, accompanied by dotted lines showing 95% confidence intervals.

To determine whether BCL11B‐induced GATA6 expression contributes to cytotoxic agent tolerance and affects prognosis, the gene and protein expressions in the HCC and surrounding liver tissues were tested by RT‐PCR, and immunohistochemical testing was conducted on the HCC tissues. RT‐PCR revealed a significant correlation between the BCL11B and GATA6 expressions in tumor and non‐tumor tissues (Figure 6A,B), and the IHC analysis supported the results in tumor tissue (Figure 6C,D). These findings indicated that GATA6 upregulation in the BCL11B‐positive HCC tissue might be related to chemosensitivity to epirubicin hydrochloride, an anthracycline.

## 4 DISCUSSION

Our study demonstrated the influence of the amplified expression of BCL11B upon prognosis. The BCL11B expression in HCC induces resistance to epirubicin hydrochloride, an anthracycline, and results in poor postoperative prognosis. The amplification of GATA6 in BCL11B overexpressed cells and BCL11B‐positive HCC tissues might be related to the anthracycline resistance. The mechanisms involved activation of anti‐apoptotic proteins, including BCL‐xL. It is important to analyze factors involved in anthracycline chemosensitivity, since for intermediate stage HCC, chemoembolization is recommended^47^, ^48^ and anthracycline has been reported to be more effective than other chemicals for chemoembolization.^49^, ^50^, ^51^ The results obtained in the current study are supported by previous study demonstrating that BCL‐xL expression is inhibited in BCL11B knockdown cells and thymocytes of BCL11B knockout mice^22^ due to transcriptional repression. A different expression changes between HLE and HepG2 cell lines might be due to the characteristics of the cells, that is, invasive and poorly differentiated HLE cells and non‐invasive and hepatoblastoma‐oriented HepG2 cells.

Furthermore, our study demonstrated the increased expression of GATA6 in BCL11B‐overexpressing cells and highly BCL11B‐expressing human tissues of HCC and surrounding liver tissues. The GATA family consists of six members (GATA1 to GATA6), which are transcriptional factors with a zinc finger structure.^52^ GATA1 to GATA3 are important for differentiation of hematopoietic stem cells and GATA4 to GATA6 are expressed in the various organs including heart, gut, pancreas, lung, and ovary.^53^, ^54^, ^55^, ^56^, ^57^ Among them, the GATA6 gene is transcribed in a pattern overlapping GATA4, and its expression is additionally found in liver tissues.^57^ Moreover, it works as an oncogenic factor in various types of tumors, including stomach,^58^ pancreatic,^41^, ^42^, ^43^ and colorectal cancers^44^, ^45^ and HCC.^46^

Recently, the oncogenic role of GATA6 on HCC has been reported in an in vitro study to counter the tumor‐suppressive effect of miR‐143.^59^ Interestingly, GATA4 and GATA6 have been reported to contribute to cardiac hypertrophy when overexpressed in cardiomyocytes, interacting with numerous cofactors under complex mechanisms.^52^ Such mechanisms involve anti‐apoptotic signal activation, supported by the findings that overexpression of GATA4 or GATA6 attenuates cardiac muscle cell apoptosis induced by anthracyclines.^60^ In addition, GATA4 has been reported to positively regulate anti‐apoptotic protein BCL‐2.^61^ These findings support the results of the present study, which indicated that GATA6 expression in hepatocytes may positively regulate BCL‐xL and BCL‐2 proteins to attenuate the apoptotic signal with epirubicin hydrochloride, and support HCC growth. The fact that BCL11B is also involved in the mechanisms of the cardiac hypertrophy^7^ suggests the potential relationship between BCL11B and GATA6.

Regarding the relationship between BCL11B and the GATA family, it has been reported that BCL11B and GATA3 collaborate to play a crucial role in T‐cell development by repressing cyclin‐dependent kinase inhibitor 2b.^62^ Moreover, it is known that the BCL11B/GATA3 complex is involved in this sequence and that BCL11B controls the GATA3‐mediated gene activation.^63^ These reports support our present results indicating the BCL11B‐associated GATA6 expression in HCC which activates anti‐apoptotic characteristics and led to the poorer postoperative prognosis than non‐BCL11B expressed HCC cases. These findings suggest that BCL11B expression in HCC might be the potential therapeutic target; however, further studies focusing on the direct relationship between BCL11B and GATA6 using Bcl11b conditional knockout mice and its contribution in HCC pathology are essential. In addition, as there are several genes up‐ and down‐regulated by the BCL11B overexpression and involved in the key roles of various oncogenic pathways (Figures 3 and 4; Tables 2 and 3), further analyses are important to explore the therapeutic target in BCL11B‐expressing HCC, which is also an important factor in various organs and cells.

The current study had several limitations: The number of cases involved is small, more than 50% of our HCC cases are associated with viral hepatitis. Therefore, based on the results obtained in this study, further analyses for larger number of HCC cases with various clinical stages and on various liver diseases including non‐alcoholic fatty liver and autoimmune liver diseases,^64^ in a multicenter study is encouraged. With the development of systemic treatment including immunotherapy and molecular target therapy for the advanced stage HCC,^65^ combination of various agents and chemoembolization is considered to improve the prognosis of HCC.^66^ Furthermore, the molecular mechanisms for these therapeutic impacts have been reported. For example, recent studies have shown the therapeutic effectiveness of capecitabine when used as a second‐line treatment after sorafenib failure by inducing T‐cell apoptosis.^67^, ^68^, ^69^ Therefore, basic and clinical studies focusing on the chemosensitivity of agents used for HCC treatment could further contribute to therapeutic advances and a safer HCC treatment.

## 5 CONCLUSIONS

Our results indicated that overexpression of BCL11B amplifies GATA6 expression in HCC in vitro and in vivo, leads to anti‐apoptotic signal activation, and induces resistance to anthracycline used in chemotherapy for HCC, which influenced the postoperative prognosis.

## AUTHOR CONTRIBUTIONS

**Hiroyuki Abe:** Conceptualization (equal); data curation (equal); formal analysis (equal); methodology (equal); validation (equal); writing – original draft (equal). **Kenya Kamimura:** Conceptualization (equal); data curation (equal); formal analysis (equal); funding acquisition (equal); project administration (equal); supervision (equal); validation (equal); visualization (equal); writing – original draft (equal). **Shujiro Okuda:** Data curation (equal); formal analysis (equal); methodology (equal); software (equal); validation (equal); writing – original draft (equal). **Yu Watanabe:** Data curation (equal); formal analysis (equal); validation (equal); writing – original draft (equal). **Jun Inoue:** Conceptualization (equal); data curation (equal); methodology (equal); supervision (equal); validation (equal); writing – original draft (equal). **Yutaka Aoyagi:** Conceptualization (equal); data curation (equal); supervision (equal); validation (equal); writing – original draft (equal). **Toshifumi Wakai:** Conceptualization (equal); formal analysis (equal); methodology (equal); supervision (equal); writing – original draft (equal). **Ryo Kominami:** Conceptualization (equal); methodology (equal); project administration (equal); supervision (equal); validation (equal); writing – original draft (equal). **Shuji Terai:** Data curation (equal); investigation (equal); project administration (equal); supervision (equal); visualization (equal); writing – original draft (equal).

## FUNDING INFORMATION

The research in the authors' laboratories has been supported in part by a grant provided by the Ichiro Kanehara Foundation.

## CONFLICT OF INTEREST STATEMENT

The authors have no conflict of interest.

## ETHICS STATEMENT

This basic and observational study protocol with the clinical samples was approved by the Ethics Committee and Institutional Review Board of Niigata University School of Medicine (approval numbers 751–716 and G2018‐0023, respectively). The study was conducted in accordance with the ethical guidance of the 1975 Declaration of Helsinki. A written informed consent was obtained from all patients for the sample collection and to publish the results based on the samples and images.

## Data Availability

The data that supports the findings of this study are available in the material of this article.
